# Reoperation in adult patients with recurrent glioblastoma: A matched cohort analysis

**DOI:** 10.1093/noajnl/vdac115

**Published:** 2022-07-13

**Authors:** Kaiyun Yang, Yosef Ellenbogen, Amanda Martyniuk, Michel Sourour, Radwan Takroni, Mohamed Somji, Emily Gardiner, Katrina Hui, Devang Odedra, Ramiro Larrazabal, Almunder Algird, Edward Kachur, Kesava Reddy, Naresh Murty, Forough Farrokhyar, Sheila K Singh

**Affiliations:** Division of Neurosurgery, McMaster University, Ontario, Canada; Division of Neurosurgery, University of Toronto, Ontario, Canada; Division of Neurosurgery, McMaster University, Ontario, Canada; Division of Neurosurgery, McMaster University, Ontario, Canada; Division of Neurosurgery, McMaster University, Ontario, Canada; Division of Neurosurgery, University of Manitoba, Manitoba, Canada; Division of Neurosurgery, McMaster University, Ontario, Canada; Division of Neurosurgery, McMaster University, Ontario, Canada; Division of General Surgery, University of Manitoba, Manitoba, Canada; Department of Psychiatry, University of Toronto, Ontario, Canada; Department of Radiology, McMaster University, Ontario, Canada; Department of Radiology, McMaster University, Ontario, Canada; Division of Neurosurgery, McMaster University, Ontario, Canada; Division of Neurosurgery, McMaster University, Ontario, Canada; Division of Neurosurgery, McMaster University, Ontario, Canada; Division of Neurosurgery, McMaster University, Ontario, Canada; Department of Surgery, Department of Health, Evidence, Impact, McMaster University, Hamilton, Ontario, Canada; Division of Neurosurgery, McMaster University, Ontario, Canada

**Keywords:** glioblastoma, overall survival, recurrence, reoperation

## Abstract

**Background:**

Despite maximal safe cytoreductive surgery and postoperative adjuvant therapies, glioblastoma (GBM) inevitably recurs and leads to deterioration of neurological status and eventual death. There is no consensus regarding the benefit of repeat resection for enhancing survival or quality of life in patients with recurrent GBM. We aimed to examine if reoperation for GBM recurrence incurs a survival benefit as well as examine its complication profile.

**Methods:**

We performed a single-center retrospective chart review on all adult patients who underwent resection of supratentorial GBM between January 1, 2008 and December 1, 2013 at our center. Patients with repeat resection were manually matched for age, sex, tumor location, and Karnofsky Performance Status (KPS) with patients who underwent single resection to compare overall survival (OS), and postoperative morbidity.

**Results:**

Of 237 patients operated with GBM, 204 underwent single resection and 33 were selected for repeat surgical resections. In a matched analysis there was no difference in the OS between groups (17.8 ± 17.6 months vs 17 ± 13.5 months, *P* = .221). In addition, repeat surgical resection had a higher rate of postoperative neurological complications compared to the initial surgery.

**Conclusions:**

When compared with matched patients who underwent a single surgical resection, patients undergoing repeat surgical resection did not show significant increase in OS and may have incurred more neurological complications related to the repeat resection. Further studies are required to assess which patients would benefit from repeat surgical resection and optimize timing of the repeat resection in selected patients.

**Key Points**

- In a matched retrospective analysis, patients who had repeat resection for recurrent GBM did not have an increased OS compared to those who had a single resection.
- Multiple surgical resections were associated with worse neurological outcomes.

**Importance of the Study**

GBM is the most common primary brain malignancy in adults. Surgical management of these lesions is the mainstay of diagnosis and treatment. However, despite maximal safe cytoreductive surgery and postoperative adjuvant therapies, GBM is an incurable disease with a grave prognosis. There is no consensus regarding the benefit of repeat resection for enhancing survival or quality of life in patients with recurrent GBM. This single-center retrospective study of 237 patients with GBM found that repeat surgical resection did not offer a survival benefit when controlling for covariates such as age, sex, KPS, and extent of resection. Though there is a need for larger prospective studies on this topic, this study provides valuable insight into the utility of repeat resection for recurrent GBM and underscores the importance of controlling for selection bias in future studies as it can significantly overestimate the effect of repeat intervention as seen in our unmatched analysis.

Glioblastoma (GBM) is the most common primary brain malignancy in adults.^1^ Despite, maximal safe cytoreductive surgery and postoperative chemotherapy and radiotherapy, the mean overall survival (OS) remains 12–15 months from initial diagnosis.^2,3^ Due to the invasive and infiltrative nature of this heterogeneous tumor, GBM inevitably recurs and leads to further neurological compromise. Repeat resection is a potential treatment option for patients who present with tumor regrowth and is offered in 10%–30% of patients in cases of recurrence.^4^ Although several studies suggest that repeat resection prolongs OS and progression-free survival (PFS),^5,6^ other investigators argue that repeat resection has limited impact on clinical course.^7–9^ In addition, there is limited consensus on which patients should be offered repeat resection and which metrics are predictors of benefit either in OS, PFS, or Health-Related Quality of Life measures.

The current literature is lacking data on the safety of the repeat operation and its impact on patient’s postoperative functional status and quality of life, beyond prolongation of survival alone. A significant challenge in investigating this patient population is selection bias, in that surgical reoperation is typically offered only to those patients who are deemed to be better surgical candidates and are thus more likely to experience benefits of surgery. Therefore, direct comparisons without matching or controlling for preoperative predictors of success can lead to an overestimation of the benefits of repeat resection. To address these questions, we retrospectively reviewed our single-center experience, and undertook a matched cohort study to specifically examine patient morbidity, postoperative neurological and functional status, and quality of life among patients undergoing repeat surgical resection for recurrent GBM.

## Methods

### Patient Population

We conducted a retrospective cohort study on all adult patients who underwent resection of primary and recurrent supratentorial GBM at our center between January 1, 2008 and December 1, 2013. Approval from the institutional research ethics board [Hamilton Integrated Research Ethics Board (HIREB)] was obtained. As such, all methods were performed in accordance with the relevant guidelines and regulations. Due to the retrospective nature of the study, the need for formal consent was waived by the HIREB. We excluded pediatric patients (age <18 years), adult patients who underwent biopsies of supratentorial GBM and those with infratentorial GBM and incomplete medical records.

### Patient Baseline and Treatment Variables

We reviewed the clinical notes from neurosurgery and multidisciplinary neuro-oncology clinics and examined baseline patient characteristics, including age, sex, presenting symptoms, tumor volume, extent of resection, and Karnofsky Performance Status (KPS). Volumetric analysis was performed on the contrast-enhancing portion of the tumor on preoperative and postoperative magnetic resonance imaging (MRI). The extent of tumor resection was determined by comparing a 24–48 h postoperative MRI to that of preoperative imaging. All patients were operated on in a single institution by four neurosurgeons. There were no significant differences in surgical technique; stereotactic neuro-navigation was routinely used. In addition, all patients were under general anesthetic during the operation. Surgical adjuncts such as neuromonitoring, 5-aminolevulinic acid and indocyanine green were not used.

All patients were followed by neurosurgery and neuro-oncology teams and underwent postoperative chemotherapy and radiotherapy (Stupp protocol) after initial resection of *de novo* GBM.^2^ Recurrent tumors were typically discovered on routine postoperative MRI that was performed at 3-month intervals following surgery, or at the time of repeat imaging due to symptom progression (eg worsening headaches, weakness, or new neurological deficits). All cases were discussed at a multidisciplinary neuro-oncology clinic, which consisted of neurosurgeons, neuro-oncologists, and radiation oncologists. Repeat operations were considered for patients with either significant radiographic occurrence or clinical decline due to tumor progression.

### Patient Outcomes

The primary outcome was OS following initial surgical resection. The date of death was obtained from the database maintained by Cancer Care Ontario. Secondary outcomes included PFS, postoperative functional status as measured by KPS, as well as morbidity and mortality associated with surgical resection. We recorded postoperative complications including cerebrospinal fluid leak, surgical cavity hematoma, meningitis, stroke, seizure, perioperative deep vein thrombosis (DVT), pulmonary embolism (PE), and death.

### Patient Matching

Direct comparison of survival and other surgical outcomes between patients who underwent single and repeat resection suffers from bias, as patients who undergo repeated resection typically have favorable characteristics that may predispose them to longer survival irrespective of the operation. Therefore, attempts were made to create a matched cohort that aims to eliminate this bias. To do this, each case was manually matched with a control for age, gender, tumor location, extent of resection, and KPS (1:1 ratio). For age, the window of ±2 years was used. The manual matching process was chosen in consultation with a biostatistician (FF) due to the small number of cases with repeated resection.

### Statistical Analysis

Descriptive statistics were calculated and reported as mean and standard deviation for continuous variables and count and percentage for categorical variables. Paired Student’s *t*-test and Wilcoxon signed rank test (nonparametric) were used to compare outcomes in the matched and unmatched cohorts. Survival analysis was conducted using the Kaplan–Meier estimator with the use of two-sided log-rank statistics. Statistical analyses were performed using STATA (Stata Statistical Software: Release 13. College Station, TX). The threshold for accepting statistical significance was set *a priori* at *α* = 0.05.

## Results

### Patient Demographics of Overall Cohort

We identified 237 patients who underwent surgical resection of GBM during the study time period. Baseline characteristics are summarized in Table 1. Overall, 204 (86.1%) patients underwent a single resection and 33 (13.9%) patients underwent at least one repeat resection for recurrence of their GBM. The cohort included 98 females and 139 males. The single resection cohort was significantly older than the repeat resection cohort (64.1 ± 10.7 years vs 54.7 ± 14.0 (*P* = .001), and almost three-quarters (72.7%) of the patients were male (Table 1). The most common presenting symptoms were headache (*n* = 110, 53.1%), followed by hemiparesis (*n* = 85, 41.1%), and seizure (*n* = 17, 8.3%). The median time to OS time for the 204 patients who underwent single resection was 8.1 (95% CI 6.2–10.0) months and for repeat resection was 16.7 (95% CI 15.3–18.2) months (*P* = .018).

**Table 1.** Descriptive analysis of baseline statistics for unmatched single and repeat resection

Patient characteristics	*N* (237)	Single resection (*N* = 204)	Repeat resection (*N* = 33)	*P*
**Age (yrs.; *N*** ^a^ **= 226)**				
		64.1 ± 10.7	54.7 ± 14.0	.001*
**Sex (*N*** ^a^ **= 237)**				
**Male**	139	115 (56.4)	24 (72.7)	.077
**Female**	98	89 (43.6)	9 (27.3)	
**Location (*N*** ^a^ **= 231)**				
**Left hemisphere**	104	90 (45.5)	14 (42.4)	.534
**Right hemisphere**	121	102 (51.5)	19 (57.6)	
**Bilateral/corpus callosum**	6	6 (3.0)	0 (0.0)	
**Presenting symptoms** ^b^ **(*N*** ^a^ **= 207)**				
**Headache**	110	92 (52.9)	18 (54.5)	.572
**Hemiparesis**	85	76 (43.7)	9 (27.2)	.084
**Seizure**	17	6 (3.5)	11 (33.3)	<.01
**Extent of resection (*N*** ^a^ **= 162)**				
**GTR**	83	79 (51.3)	4 (50.0)	.943
**STR**	79	75 (48.7)	4 (50.0)	

^a^ *N* represents the number of patients for which the data point was available. Total *N* = 237. Percentages are derived using the *N* of available data points per cohort (single or repeat) as the denominator.

^b^Note some patients presented with multiple symptoms and thus may be represented in each of the rows.

*indicates *P* < 0.05

### Patient Demographics of Matched Cohort

Subgroup analysis was conducted by matching 33 patients who underwent repeat resection with patients with similar preoperative characteristics who had a single resection. The matched single and repeat resection cohorts were similar with respect to age, sex, tumor location, presenting symptoms, extent of resection, and KPS (Table 2). In the repeat resection cohort, the average time from initial resection to repeat resection was 10.4 ± 4.7 months. In addition, two patients in the repeat resection cohort had an additional (third) resection at a later timepoint.

**Table 2.** Descriptive analysis of baseline statistics for matched single and repeat resection

Patient characteristics	Single resection (*N* = 33)	Repeat resection (*N* = 33)	*P*
Age (yrs)			
	58.6 ± 9.97	**54.7 ± 14.0**	.065
Sex			
Male	24 (72.7)	24 (72.7)	1.000
Female	9 (27.3)	9 (27.3)	
Location			
Left hemisphere	16 (48.5)	**14 (42.4)**	1.000
Right hemisphere	17 (51.5)	**19 (57.6)**	
Bilateral/corpus callosum	0 (0)	0 (0.0)	N/A
Presenting symptoms			
Headache	20 (60.6)	18 (54.5)	.655
Hemiparesis	11 (33.3)^a^	**9 (27.2)**	.564
Seizure	9 (27.3)	11 (33.3)	.617
Extent of resection			
GTR	18 (54.5)	18 (54.5)	1.000
STR	15 (45.5)	15 (45.5)	
Repeat resection			
Second	N/A	33 (100)	N/A
Third	N/A	2 (6)	

^a^Missing 1 patient’s data due to incomplete charting.

Of note, for the 33 pairs of matched patients, 16 pairs had tumors located in the left hemisphere. In terms of location, 11 pairs of patients harbored tumors in the frontal lobe, 17 in the temporal lobe, 4 in the parietal lobe, and 1 in the occipital lobe. All tumors were superficially located within 1 cm of cortical surface. This may suggest a selection bias for superficially located tumors in relatively noneloquent regions in high-functioning patients to be considered for repeat resections, which influenced decision-making favoring more aggressive surgical management. Patient and surgeon preference also factored into decision-making for repeat operation.

### Patient Outcomes of the Matched Cohort

**Figure 1.**
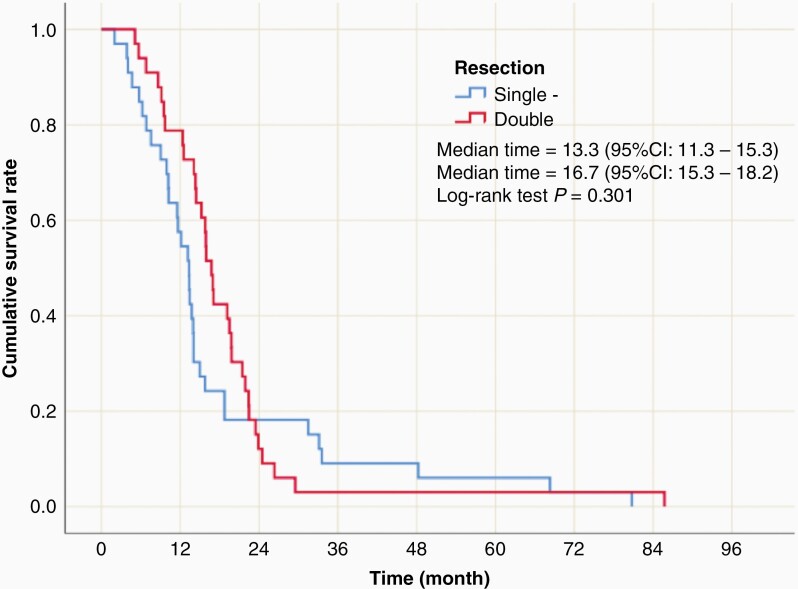
Kaplan–Meier survival curves revealed no significant difference in median survival in matched patient cohorts. The median survival time was 13.3 months (95% CI 11.3–15.3) in the single resection cohort and 16.7 months (95% CI 15.3–18.2) in the repeat resection group (*P* = .301).

In the matched cohorts, Kaplan–Meier survival analysis revealed no significant difference in mean or median survival between the two groups. The median survival time was 13.3 months (95% CI 11.3–15.3) in the single resection cohort and 16.7 months (95% CI 15.3–18.2) in the repeat resection group (*P* = .301) (Figure 1). In the patients who underwent repeat resections, the median OS from the time of their second surgery was 5.0 (95% CI 2.6–7.0) months.

We observed a significant decline in patients’ functional status at the time of recurrence in the matched cohorts. For those who underwent a single resection, the majority presented with worse neurological function (KPS ≤ 50), and significant medical morbidities. Therefore, patients and families were less interested in pursuing further surgical resection, and surgeons were less inclined to recommend it due to its limited benefits. For the patient who underwent repeat resection, mean pre-2nd OR KPS was 69 ± 17.1, and mean post-2nd OR KPS was 64 ± 23.7, with a mild, insignificant drop of 5.1 in KPS following the second operation (*P* = .051). However, the mean post-2nd OR KPS was significantly lower than that following initial resection (83 ± 10.2 vs 64 ± 23.7, *P* < .0001) (Table 3). This decline in postoperative KPS following the second operation occurred because of a combination of neurological decline caused by disease progression and occurrence of new neurological deficits after the second operation (7 patients, 21.2%).

**Table 3.** Outcomes for matched single and double resection

	Single resection (*N* = 33)	Repeat resection (*N* = 33)		P
		First operation	Second operation	
**Median survival (months)**	13.3 (11.3–15.3)	16.7 (15.3–18.2)	5.0 (2.6–7.0)	.301^a^
**Mean Postoperative KPS (*N* = 33)**	80.3 (76.7–834.0)	83.0 (79.4–86.6)	64.2 (55.8–72.6)	.027^b^

^a^Comparing median time to survival from first operation.

^b^Comparing KPS after first operation. The values inside the brackets present 95% confidence interval.

The comparison of complications between the groups is summarized in Table 4. Briefly, the neurological and nonneurological complications between the two groups occurred at similar frequencies during the first operation. In the single resection group, three patients (9.1%) had a neurologic complication (one with seizure and two with cognitive impairment) and in the repeat resection group one patient (3.0%) had a surgical cavity hematoma. In addition, the nonneurological complications were comparable between the two groups occurring in six patients (18.2%) in the single resection cohort and seven patients (21.2%) in the repeat resection cohort. However, the rate of neurological complications experienced during the second operation of the repeat resection cohort was markedly higher consisting of 12 patients (36.3%) with 7 (21.1%) patients experiencing a new focal neurological deficit. In contrast, the second operation did not yield an increase in the rate of nonneurological complications. The complications have also been summarized using the Clavien–Dindo classification system (Table 5). The same trend is demonstrated with an increased frequency of higher-grade complications noted in the second operation.

**Table 4.** Frequency of complications in patients for matched single and double resection

	Single resection *N* = 33	Repeat resection (*N* = 33)	
		First operation	Second operation
**Neurological**	**3 (9.1)**	**1 (3.0)**	**12 (36.4)**
New focal neurological deficit	0	0	7 (21.2)*
Major vascular infarct	0	0	1 (3.0)
Surgical cavity hematoma	0	1 (3.0)	1 (3.0)
CNS infection	0	0	1 (3.0)
Hydrocephalus	0	0	1 (3.0)
Seizure	1 (3.0)	0	0
Cognitive impairment	2 (6.1)	0	1 (3.0)
**Nonneurological**	**6 (18.2)**	**7 (21.2)**	**4 (12.1)**
UTI	1 (3.0)	0	0
DVT	1 (3.0)	4	0
PE	0	1 (3.0)	0
Sepsis	1 (3.0)	0	1 (3.0)
Cardiac arrhythmia	0	2	1 (3.0)
Superficial surgical site infection	0	0	1 (3.0)
Long bone fracture	0	0	1 (3.0)
Pneumonia	1 (3.0)	0	0
Prolonged ICU stay (>3d)	2 (6.1)	0	0

^a^Deficits included ataxia (*n* = 2), word-finding difficulties (*n* = 2, motor weakness (*n* = 1), visual field deficit (*n* = 1), and visual acuity deficit (*n* = 1).

**Table 5.** Clavien–Dindo classification of complications in patients for matched single and repeat resection

	Single resection	Repeat resection (first operation)	Repeat resection (second operation)
**Grade 1**	2 (6.1)	1 (3.0)	1 (3.0)
**Grade 2**	4 (12.1)	5 (15.2)	1 (3.0)
**Grade 3**	0	0	0
**Grade 4**	3 (9.1)	0	7 (21.2)
**Grade 5**	0	0	0

## Discussion

The utility of repeat surgical resection for recurrent GBM is an area of active investigation and there is no consensus on which patients would benefit from repeat surgery. The present retrospective study assessed the outcomes of patients with GBM who underwent a single or repeat resection at a single institution with extended follow-up from time of diagnosis until the time of death. Though the unmatched cohort showed a significant increase in OS, this effect was lost when the patients were matched to have similar baseline characteristics. Despite the lack of molecular data, we matched patients based on age, sex, tumor location, extent of resection, and functional status post initial surgical resection, for a comparable baseline. All patients in the match cohorts subsequently received the same standardized adjuvant therapy with Stupp protocol. Significant functional decline was observed at the time of recurrence in both groups. Despite relatively reasonable KPS at the time of recurrence and maintained postoperative functional status in the patients who underwent repeat resection, there was no significant survival benefit associated with repeat operations. In addition, we found that repeat surgical resection carried a higher risk of postoperative neurological complications and overall higher-grade Clavien–Dindo complications related to the procedure.^10^

Prior studies that have assessed surgical outcomes from repeat resection for GBM have had mixed results.^8,11,12^ For example, in a study by Chaichana *et al.* consisting of 578 patients, 224 patients had at least one repeated resection. This study found that patients who underwent a repeat resection had a prolonged OS without added risk of postoperative complications. The patients who had repeated resections were significantly younger although the KPS was not different between the groups with a median score of 80 in both groups.^5^ Another study by Goldman *et al.* consisted of 163 patients, 89 of whom had repeat resections for recurrent GBM, and found that OS was not different between the two groups.^9^ In their study, patients who underwent repeat resection were younger with higher KPS and greater extent of resection at initial surgery. Another study by Filippini *et al.* consisted of 676 patients, 25% of whom had repeat surgical resection with no improvement in OS.^13^ However, all of these studies were limited by lack of baseline equivalence between the groups resulting in selection bias that limits the conclusions that can be drawn from these analyses.

Another important consideration in determining whether repeat surgical resection has a survival benefit in the timing of repeat surgery. In studies where the timing of surgery is not taken into consideration, the effect of the surgery on survival may be over-estimated. Indeed, in a meta-analysis of 21 studies consisting of 8,360 patients, Zhao *et al.* found that the survival benefit was over-estimated when repeat surgery was considered a fixed covariate.^14^ Goldman *et al*. observed a similar finding in that repeat operation was found to have a survival benefit when timing was not considered, but the effect dissipated when the timing was added to the analysis.^9^ This highlights the need to include timing in the analysis. Though our sample size precludes a robust analysis on the impact of timing of the second operation, the majority of our patients underwent a repeated resection within 6 months after the initial surgery. In addition, our finding of no survival benefit in matched cohorts is in agreement with these other studies.

The limitations of this study are related to its single-center and retrospective nature, which introduces several limitations including selection bias, sampling bias, incomplete data particularly lack of molecular marker such as IDH mutation, and MGMT promoter methylation prior to revision of WHO grading and lack of data regarding physical presentation at time of recurrence,^15,16^ adjuvant treatment heterogeneity after recurrence, and lack of direct comparison between the two cohorts. The choice to do repeat surgery was not standardized and subject to the surgeon’s preferences, with a preference to repeat resections for superficially located tumors in relatively noneloquent locations with low-risk profiles. The reasons for nonoperative management at time of recurrence for the matched cohort who did not undergo repeat resection are multifactorial. In the single resection group, some of these tumors behaved more aggressively, with faster and more aggressive recurrence, recurrence in more eloquent regions making meaningful resection with survival benefits more challenging, and/or caused more neurological declines and worse KPS in the absence of a sizable recurrence. In addition, there were also considerations of patients’ general health and medical comorbidities. All these factors, particularly tumor behavior, have influenced patient and family wishes, as well as surgeon preferences and recommendations. Direct comparison of patients who proceeded with a second operation to those who declined it at the time of recurrence with similar characteristics would also help strengthen the study. However, the sample size of 33 patients in each group is underpowered for this purpose and to detect minor differences in OS. Health-Related Quality of Life measures were not collected, and this may be an indication to offer repeat surgery even if it does not confer a survival benefit. For example, in patients with high tumor burden or dependence on steroids and have good functional status, there may be a benefit from repeat surgery in symptom management. Other temporal data points such as neurologic symptoms and KPS as a function of time were not available due to incomplete documentation, limiting the matched analysis to only comparing OS. Another limitation is the study time, which is approximately 10 years old (2008–13).

Despite these limitations, this is a large dataset and the case-control design is unique. Our research question remains unanswered in the literature and there is still equipoise around the question. Despite the data gathering ending in 2016, there has been no further advance on the management of these patients and the present study is in keeping with the previous literature showing that reoperation does not confer a significant survival benefit.^5,11,13,17^ In addition, our study demonstrated a potential increase in complications related to repeat surgical resection. This may be due to the inherent challenges of repeated surgical resections due to scar tissue and distorted surgical anatomy. This finding is congruent with other reports of increased surgical morbidity associated with repeat resection. Ringel *et al.* found a modest increase in surgical complications in a cohort of 503 patients who underwent repeat resection for recurrent GBM.^7^ However, the evidence is mixed, and others have reported similar rates of complications compared with the initial resection.^18^

Although analysis of our total patient cohort reveals an apparent survival benefit in patients undergoing repeat surgical resection for GBM recurrence, an attempt to control for surgical selection bias through a matched cohort study revealed no significant difference in survival between matched patients undergoing single resection and repeat resections. The question of when to offer repeat surgical resection for recurrent GBM remains unanswered. Though there may not be an overall significant survival benefit, the timing of the procedure and subset of patients operated on are critical in maximizing the benefit of repeat surgical resection. Thus, there is a need for high-quality studies to prospectively analyze these patient populations and quantify the benefits seen with the time of reoperation taken into consideration in the analysis. In addition, the molecular subtypes of the tumor may also play a role in whether repeat surgical resection is beneficial, a factor relatively unexplored to date. Prior studies have found that IDH-1 and MGMT promoter methylation status influence treatment response and survival in patients with GBM, and this should be analyzed in the context of response to repeat surgical resection as well.

## Conclusion

Our study found that in patients with recurrent GBM, repeat surgery tends to be offered to patients with high functional status and superficial tumors located in relatively noneloquent regions. Repeat operation did not confer a survival benefit in a matched cohort analysis between patients who had single vs repeat surgery and had higher rates of neurological complications. Further work is required to assess which patients would benefit from repeat surgical resection.
